# Decoy receptor 2 mediates the apoptosis-resistant phenotype of senescent renal tubular cells and accelerates renal fibrosis in diabetic nephropathy

**DOI:** 10.1038/s41419-022-04972-w

**Published:** 2022-06-03

**Authors:** Jia Chen, Ke-hong Chen, Li-ming Wang, Jia Luo, Quan-you Zheng, Ya-ni He

**Affiliations:** 1 Department of Nephrology, Daping Hospital, Army Medical University, Chongqing, 400042 China; 2 Department of Nephrology and Urology, The 958th Hospital, The First Affiliated Hospital, Army Medical University, Chongqing, 400020 China

**Keywords:** Chronic kidney disease, Interstitial disease

## Abstract

Apoptotic resistance leads to persistent accumulation of senescent cells and sustained expression of a senescence-associated secretory phenotype, playing an essential role in the progression of tissue fibrosis. However, whether senescent renal tubular epithelial cells (RTECs) exhibit an apoptosis-resistant phenotype, and the role of this phenotype in diabetic nephropathy (DN) remain unclear. Our previous study was the first to demonstrate that decoy receptor 2 (DcR2) is associated with apoptotic resistance in senescent RTECs and renal fibrosis. In this study, we aimed to further explore the mechanism of DcR2 in apoptosis-resistant RTECs and renal fibrosis in DN. DcR2 was co-localized with fibrotic markers (α-SMA, collagen IV, fibronectin), senescent marker p16, and antiapoptotic proteins FLIP and Bcl2 but rarely co-localized with caspase 3 or TUNEL. DcR2 overexpression promoted renal fibrosis in mice with streptozotocin (STZ)-induced DN, as evidenced by augmented Masson staining and upregulated expression of fibrotic markers. DcR2 overexpression also enhanced FLIP expression while reducing the expression of pro-apoptotic proteins (caspases 8 and 3) in senescent RTECs, resulting in apoptotic resistance. In contrast, DcR2 knockdown produced the opposite effects in vitro and in vivo. Moreover, quantitative proteomics and co-immunoprecipitation experiments demonstrated that DcR2 interacted with glucose-related protein 78 kDa (GRP78), which has been shown to promote apoptotic resistance in cancer. GRP78 exhibited co-localization with senescent and antiapoptotic markers but was rarely co-expressed with caspase 3 or TUNEL. Additionally, GRP78 knockdown decreased the apoptosis resistance of HG-induced senescent RTECs with upregulated cleaved caspase 3 and increased the percentage of apoptotic RTECs. Mechanistically, DcR2 mediated apoptotic resistance in senescent RTECs by enhancing GRP78–caspase 7 interactions and promoting Akt phosphorylation. Thus, DcR2 mediated the apoptotic resistance of senescent RTECs and renal fibrosis by interacting with GRP78, indicating that targeting the DcR2–GRP78 axis represents a promising therapeutic strategy for DN.

## Introduction

Diabetic nephropathy (DN) occurs in 20–40% of patients with diabetes and is the leading cause of end-stage renal disease [1, 2]. Accumulating evidence suggests that renal fibrosis is an essential mediator in the progression of DN [3, 4]. Although renal fibrosis scores are positively correlated with renal dysfunction, which is a powerful predictor of prognosis in patients with DN [5], the mechanisms underlying this association remain to be fully clarified.

Cellular senescence, in which stable cell cycle arrest is triggered by repetitive cellular mitoses and various stressors, plays key roles in various physiological and pathological processes [6, 7]. The beneficial or deleterious effects of senescent cells depend on whether the senescent state is transitory or persistent [6–9]. While transient senescence plays a protective role in organogenesis and tissue homeostasis during embryonic development, the persistence of senescent cells has been increasingly recognized as a powerful driver of several diseases, including diabetes mellitus, cancer, and cardiovascular disease [10, 11]. The detrimental effects of persistent cellular senescence are closely related to the senescence-associated secretory phenotype (SASP), which is characterized by multiple factors that contribute to chronic inflammation, adverse tissue remodeling, and fibrosis [12]. Several studies have indicated that senescent cells contribute to a pro-fibrotic milieu via the SASP, and the presence of senescent renal tubular epithelial cells (RTECs) has been positively correlated with renal fibrosis in mouse models, patients with DN, and renal transplant recipients [13–16]. Furthermore, both silencing of p16^INK4a^ expression and clearance of p16-positive senescent RTECs have been shown to significantly attenuate renal fibrosis and increase survival [17–19]. These findings suggest that RTEC senescence is associated with the progression of renal disease and highlight its critical role in the pathogenesis of renal fibrosis [8, 20].

In addition to growth arrest, increased resistance to apoptosis is a significant functional hallmark of senescent cells [21, 22]. Apoptotic resistance not only inhibits the elimination of senescent cells but also leads to their accumulation during the aging process [21, 22], which may promote disease progression and shorten life expectancy via sustained secretion of the SASP [19]. Several studies have reported that targeting the elimination of senescent RTECs represents a promising strategy for treating kidney diseases [23, 24]. Uncovering the molecular mechanisms underlying apoptotic resistance in senescent RTECs may shed light on novel strategies for the prevention and treatment of DN. Research has indicated that senescent fibroblasts exhibit downregulation of pro-apoptotic proteins (Bak and Bax) and upregulation of antiapoptotic Bcl-2 family proteins (Bcl-2, Bcl-W, and Bcl-XL), thereby contributing to the pathogenesis of pulmonary injury [25, 26]. However, the role and molecular mechanism of apoptotic resistance in senescent RTECs in DN remain unclear.

Decoy receptor 2 (DcR2), a transmembrane receptor of tumor necrosis factor (TNF)-related apoptosis-inducing ligand, is a member of the TNF receptor superfamily [27] that has been considered a tentative marker of cellular senescence [28, 29]. In our recent study, we identified DcR2 as a specific marker of senescence in RTECs, with other studies adding that DcR2 mediates renal fibrosis by enhancing senescent phenotypes in RTECs [14, 15]. Moreover, previous studies have suggested that DcR2 antagonizes the apoptotic effects induced by chemotherapy drugs, resulting in tumor progression [30, 31]. Senescent cells are also highly resistant to TNF-related apoptosis-inducing ligand-induced apoptosis via upregulation of DcR2 expression [29, 32]. Interestingly, our published data indicated that DcR2-positive senescent RTECs possessed an antiapoptotic phenotype, as evidenced by overexpression of the antiapoptotic protein FLICE-like inhibitory protein (FLIP) and little-to-no expression of caspase-3 [20]. Based on this observation, we hypothesized that DcR2 mediates apoptotic resistance in senescent RTECs, thereby promoting the development of diabetic renal fibrosis. In this study, we aimed to further explore the contribution of DcR2 to the apoptotic-resistant phenotype of senescent RTECs and the mechanisms by which they contribute to renal fibrosis in vitro and in vivo.

## Results

### DcR2 accelerates renal fibrosis in diabetic nephropathy

**Fig. 1:**
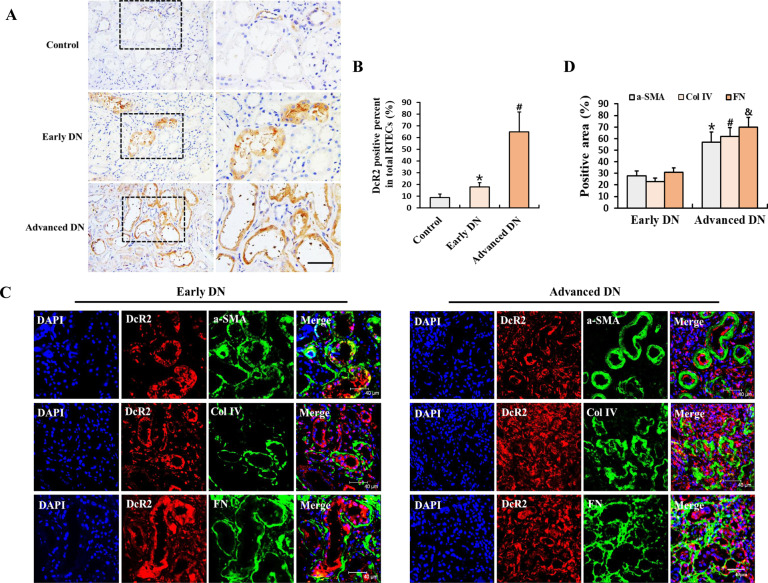
Analysis of DcR2 expression and co-localization with fibrotic markers in patients with DN. **A** Immunohistochemical staining for DcR2 in controls and patients with DN. Scale bar, 40 μm. **B** The percentage of DcR2-positive RTECs was quantified (*n* = 241). Data are expressed as the mean ± SD for each group. *P < 0.05 versus control; ^#^*P* < 0.05 versus early DN. **C** Representative immunofluorescence staining for DcR2 (red) and fibrotic markers (α-SMA, collagen IV, and fibronectin) (green) in patients with early and advanced DN. Scale bar, 40 μm. **D** Percentage of the area exhibiting fibrotic marker expression in patients with DN. */^#^/^&^*P* < 0.05 versus early DN.

First, we explored DcR2 expression in 241 tissue samples from patients with biopsy-proven DN (83 early DN and 158 advanced DN) and 18 control samples from individuals with normal kidney function using immunohistochemistry. The clinical characteristics of these participants are presented in Supplementary Table S1. DcR2 was predominantly expressed in RTECs but not in glomeruli (Fig. 1A). The percentage of DcR2-positive RTECs was clearly upregulated in tissue samples from patients with early DN and further increased in advanced DN (Fig. 1B). As shown in Fig. 1C, dramatically enhanced expression of DcR2 was observed surrounding the area exhibiting fibrotic marker expression (α-smooth muscle actin (α-SMA), collagen IV, and fibronectin) in the tubulointerstitium of both early and advanced DN. Furthermore, levels of fibrotic markers increased with the progression of DN, which was consistent with changes in DcR2 expression (Fig. 1D). These results indicate that DcR2 was overexpressed in DN and increased with the progression of DN.

**Fig. 2:**
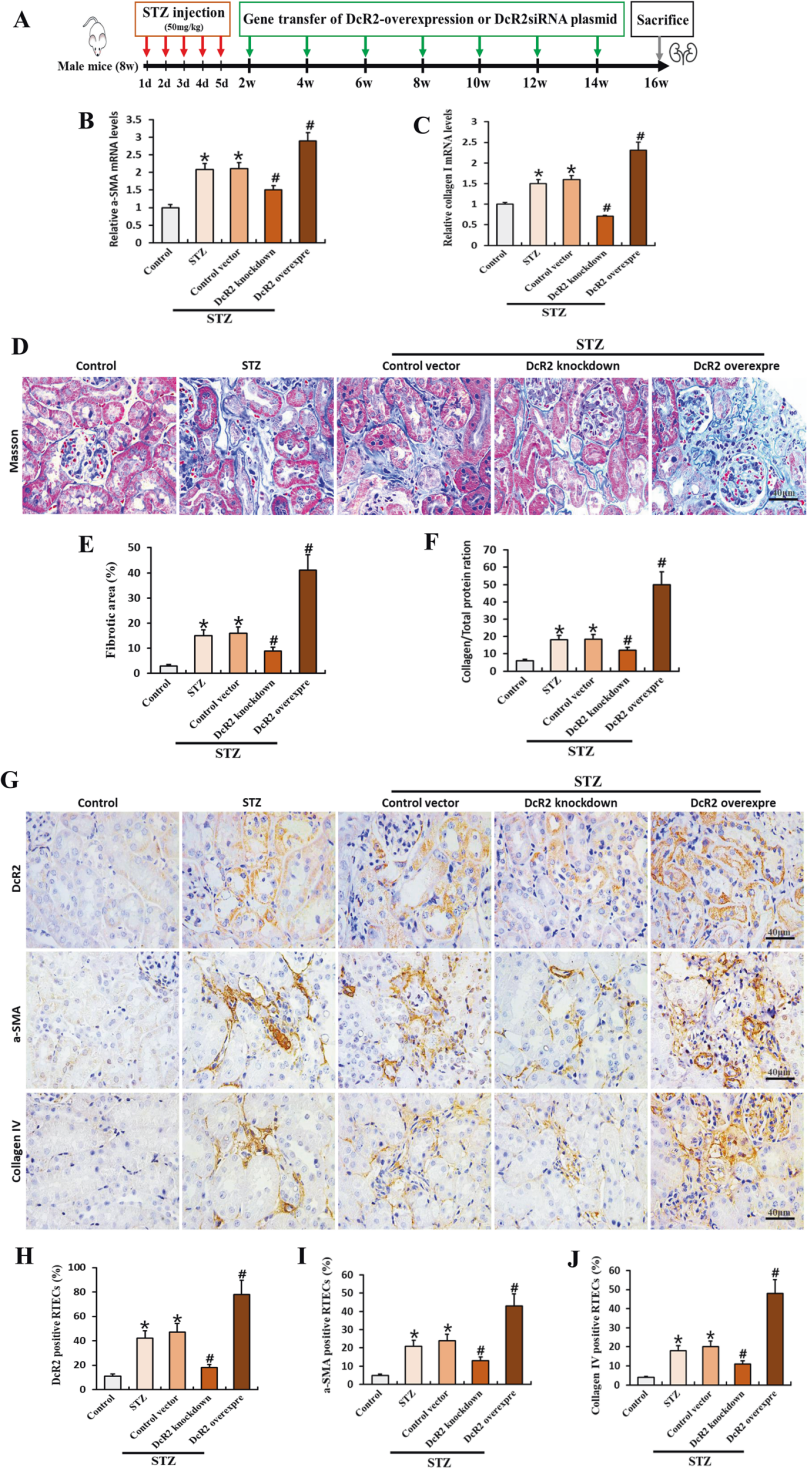
Elevated DcR2 exacerbates renal fibrosis in STZ-induced DN. **A** Experimental design. Red arrows indicate the injection of STZ (mg/kg), while green arrows indicate the transfer of pLenti-C-mGFP DcR2 overexpression or pGFP-C-shLenti DcR2-siRNA plasmids. **B**, **C** qRT-PCR results for α-SMA and collagen I mRNA levels in each group (*n* = 6). **D**, **E** Masson trichrome staining showing renal fibrosis (blue) and the percentage of fibrosis in different groups after quantitative analysis. Scale bar, 40 μm. **F** Total collagen in each group (*n* = 6). **G** Immunohistochemical staining for DcR2, α-SMA, and collagen IV. Scale bar, 40 μm. **H**–**J**. The percentage of positive RTECs was quantified (*n* = 6–7). Data are expressed as the mean ± SD for each group. *P < 0.05 versus control; ^#^*P* < 0.05 versus STZ.

To further investigate the role of DcR2 in renal fibrosis, we conducted mouse experiments in which green fluorescent protein (GFP)-DcR2 small interfering RNA (siRNA) or overexpression plasmids were injected into the kidney via the tail vein under ultrasound guidance, in accordance with established procedures (Fig. 2A) [33]. As shown in Supplementary Fig. S1A, GFP was mainly expressed in renal tubules in mice with streptozotocin (STZ)-induced DN that had been transfected with DcR2-related plasmids. Renal levels of DcR2 mRNA and protein were also significantly increased in STZ-induced DN mice transfected with DcR2-overexpression plasmid when compared with those observed in mice transfected with the control vector. In contrast, DcR2-siRNA plasmid transfection decreased DcR2 mRNA and protein levels relative to those observed in control experiments (Figs. S1B–D and 2G, H). In mice with STZ-induced DN, DcR2 overexpression promoted STZ-induced renal fibrosis, as evidenced by increased expression of α-SMA and collagen I mRNA (Fig. 2B and C), expansion of the fibrotic area in Masson staining (Fig. 2D, E), increased collagen levels (Fig. 2F), and upregulated expression of fibrotic proteins (α-SMA and collagen IV, Fig. 2G–J). In contrast, DcR2 knockdown with siRNA produced the opposite effects (Fig. 2). Collectively, these results indicate that DcR2 mediates the pathogenesis of renal fibrosis in DN.

### DcR2-positive senescent RTECs possess apoptotic resistance and SASP phenotypes in DN

**Fig. 3:**
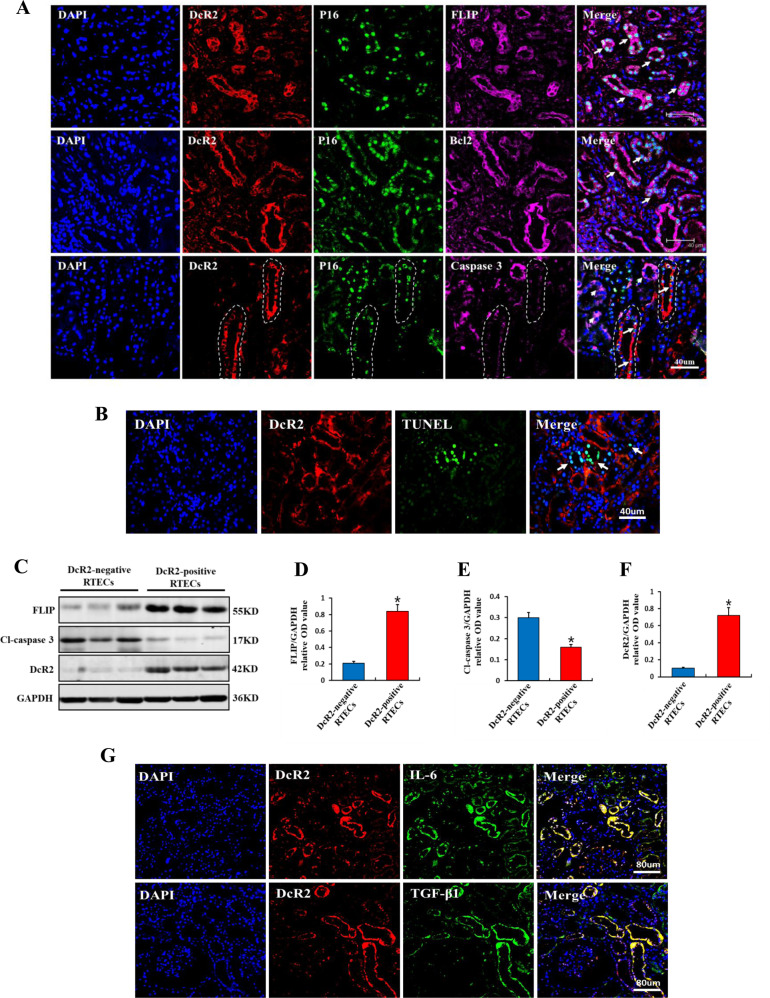
DcR2-positive senescent RTECs exhibited an apoptosis-resistant phenotype and SASP in DN samples. **A** Representative immunofluorescence staining for DcR2 (red), the senescent marker p16 (green), and markers of apoptosis resistance (FLIP and Bcl2, purple) or apoptosis (caspase 3, purple) in patients with DN. Long arrows indicate co-localization in the same tubules, while short arrows indicate cytoplasmic p16 and caspase 3-positive expression in renal tubules. White dotted circles indicate tubular areas positive for DcR2 and p16 but negative for caspase 3. Scale bar, 40 μm. **B** Representative immunofluorescence micrographs showing DcR2 and TUNEL expression (green) in STZ-DN mice. Arrows indicate the single TUNEL-positive expression in tubules. Scale bar, 40 μm. **C** Western blotting results for the expression of FLIP, cleaved caspase 3, and DcR2 in DcR2-negative and -positive RTECs. **D**–**F** Quantification of FLIP, cleaved caspase 3, and DcR2 expression in C, normalized to GAPDH (*n* = 4). *P < 0.05 versus DcR2-negative RTECs. **G** Representative graphs showing DcR2 and SASP markers (IL-6, TGF-β1) in STZ-DN mice. Scale bar, 80 μm.

Increased resistance to apoptosis is a significant functional hallmark of senescent cells [22]. In our previous study, purified DcR2-positive senescent cells exhibited an antiapoptotic phenotype characterized by increased FLIP expression [20]. To investigate whether senescent RTECs exhibit antiapoptotic phenotypes in the context of diabetes mellitus, we generated a mouse model of DN using STZ. As shown in Fig. 3A, DcR2 was co-localized with the senescent marker nuclear p16 and the antiapoptotic proteins FLIP and Bcl-2 in patients with DN. The co-localization was stronger in the first and second row than in the third, which might be related to tubulointerstitial injury resulting in relatively higher levels of DcR2 and nuclear p16. Despite the absence of co-localization (DcR2 and nuclear p16) in some tubular cells, which reflects the fact that not all senescent cells express DcR2, the co-localization of DcR2 and nuclear p16 is still strong in the third row (Fig. 3A). In contrast, nuclear p16-DcR2 positive tubular cells in the third row (long white arrows) had lost caspase 3 expression (Fig. 3A). Accordingly, DcR2 was not co-expressed with TUNEL-positive apoptotic tubular cells in STZ-DN mice (Fig. 3B). In vitro, magnetically purified DcR2-negative RTECs exhibited low FLIP expression, low DcR2 expression, and high cleaved caspase 3 expression. In contrast, DcR2-positive RTECs exhibited significantly enhanced FLIP and DcR2 expression without detectable expression of cleaved caspase 3 (Fig. 3C–F). These data support the notion that DcR2-positive senescent tubular cells exhibit low levels of apoptosis. Moreover, DcR2 was co-expressed with the SASP markers interleukin-6 (IL-6) and transforming growth factor beta (TGF-β) in STZ-DN mice (Fig. 3G). Collectively, these results clearly suggest that DcR2-positive RTECs exhibit antiapoptotic and SASP phenotypes in DN-induced renal fibrosis.

### DcR2 mediates the apoptosis-resistant phenotype of senescent RTECs

**Fig. 4:**
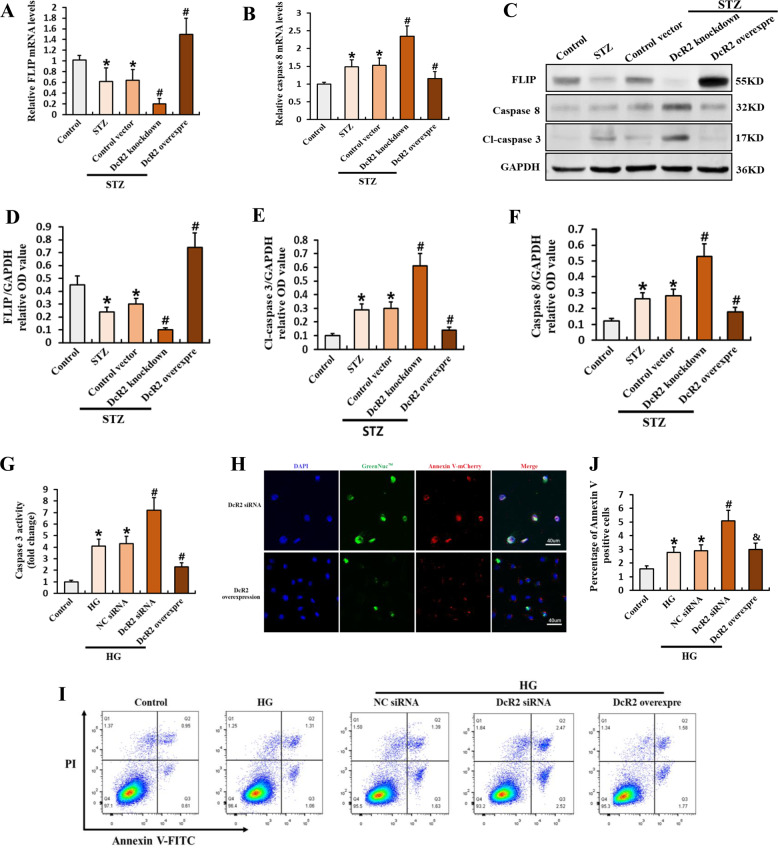
DcR2 mediated the apoptosis-resistant phenotype of senescent RTECs in vivo and in vitro. **A**, **B** qRT-PCR results for FLIP and caspase 8 mRNA levels in each group (*n* = 6). **C**. Western blotting results for the expression of FLIP, caspase 8, and cleaved caspase 3 in STZ-DN mice. **D**–**F** Quantification of FLIP, caspase 8, and cleaved caspase 3 expression in C, normalized to GAPDH (*n* = 6). **G** Caspase 3 activity in HG-induced primary RTECs (*n* = 4). **H** Representative micrographs showing the expression of caspase 3 (green) and Annexin V (red) in vitro. Scale bar, 40 μm. **I**–**J** Apoptosis rate as detected via flow cytometry and the percentage of Annexin V-positive RTECs (*n* = 6 for each group). Data are expressed as the mean ± SD for each group. *P < 0.05 versus control; ^**#**^*P* < 0.05 versus STZ/ HG; ^&^*P* < 0.05 versus HG + DcR2siRNA.

To further explore the effects of DcR2 on apoptotic resistance in senescent RTECs, we examined the antiapoptotic phenotype using STZ-DN mice. Figure 4A, B shows that DcR2 knockdown clearly decreased mRNA expression of FLIP and increased mRNA expression of caspase 8; however, DcR2 overexpression reversed these effects. Similar results were obtained for anti- and proapoptotic protein levels in the western blot analysis (Fig. 4C–F). Consistent with the in vivo results, DcR2 siRNA remarkably increased levels of high glucose (HG)-induced apoptosis in primary mouse RTECs. In these experiments, increased apoptosis was accompanied by upregulated caspase 3 activity (Fig. 4G), increased co-staining of caspase 3 and Annexin V (Fig. 4H), and an increase in the percentage of apoptotic cells (Fig. 4I, J). However, transfection of the DcR2-overexpression plasmid produced the opposite effects (Fig. 4G–J). Accordingly, these results suggest that DcR2 mediates the antiapoptotic phenotype of senescent RTECs both in vivo and in vitro.

### Proteomic identification of GRP78 is a novel DcR2-interacting protein

**Fig. 5:**
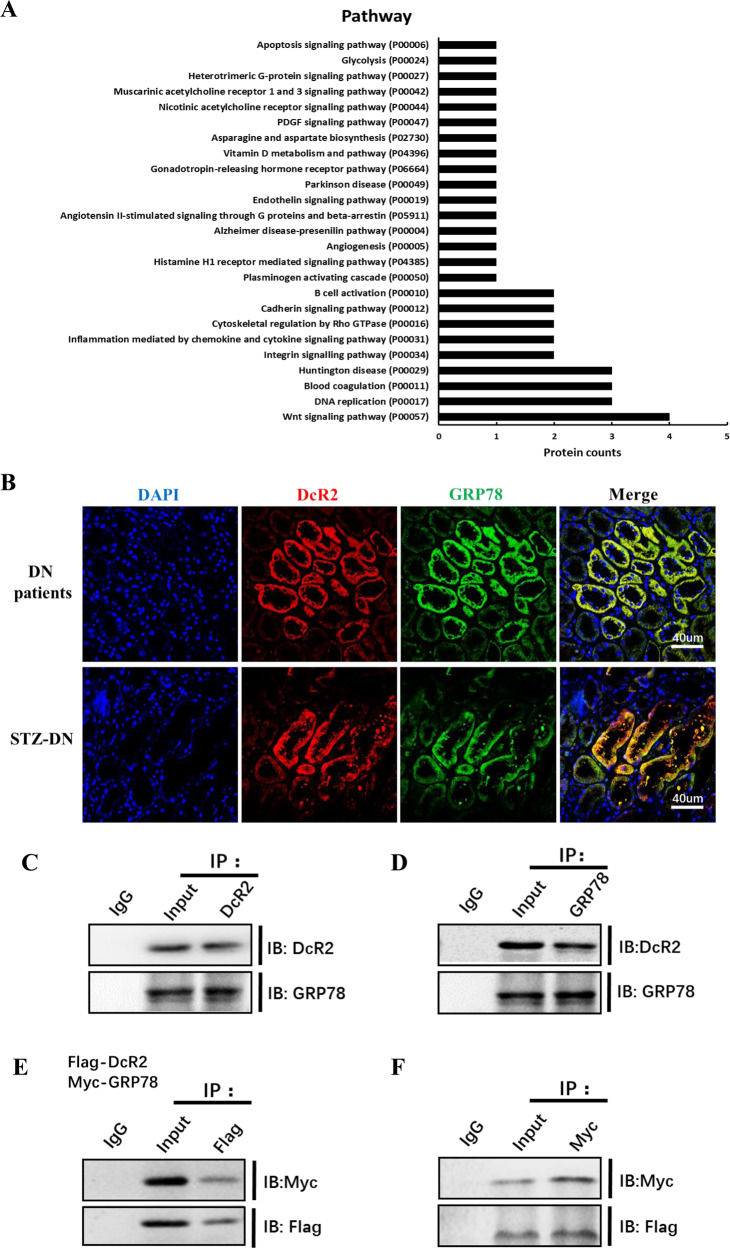
Proteomic identification, functional analysis, and validation of DcR2-interacting proteins. **A** Pathway analysis for differentially expressed proteins in patients with DN versus controls. **B** Representative immunofluorescence staining for DcR2 (red) and GRP78 (green) in renal tissues from patients with DN and STZ-induced DN models (*n* = 6 for each group). Scale bar, 40 μm. **C**, **D** Reciprocal co-immunoprecipitation showing the interaction of DcR2 and GRP78 in STZ-DN (*n* = 6). **E**, **F** Immunoprecipitation assays of RETCs co-transfected with Flag-DcR2 or Myc-GRP78, followed by immunoblotting with anti-Myc or anti-Flag, respectively (*n* = 3).

Liquid chromatography with tandem mass spectrometry (LC-MS/MS) quantitative proteomic coupled with immunoprecipitation (IP) was used to analyze DcR2-interacting proteins in renal biopsies obtained from patients with DN. Following large-scale identification and functional categorization of differentially expressed proteins, we identified 135 proteins that exhibited >2.0-fold changes in expression in patients with DN vs. healthy controls (*p* < 0.05), in accordance with our recently published study [15]. Additionally, KEGG analysis revealed that these differentially expressed proteins were closely related to several pathways, including those involved in apoptosis, glycolysis, G-protein interactions, and Wnt signaling (Fig. 5A). We focused on glucose-related protein 78 kDa (GRP78) in subsequent experiments given its associations with cellular senescence and apoptotic resistance in previous studies [34–36].

We conducted dual immunostaining experiments to confirm the interaction between DcR2 and GRP78, observing co-localization of DcR2 and GRP78 in RTECs in both patients with DN and STZ-DN mice (Fig. 5B). This interaction was also observed in co-IP experiments in STZ-DN mice (Fig. 5C, D). Moreover, Flag or Myc was immunoprecipitated with Flag-DcR2 or Myc-GRP78, followed by immunoblotting with anti-Myc or anti-FLAG, respectively (Fig. 5E, F). These results verified that GRP78 is a novel DcR2-interacting protein in DN.

### DcR2 interacts with GRP78 to mediate the apoptosis-resistant phenotype of senescent RTECs

**Fig. 6:**
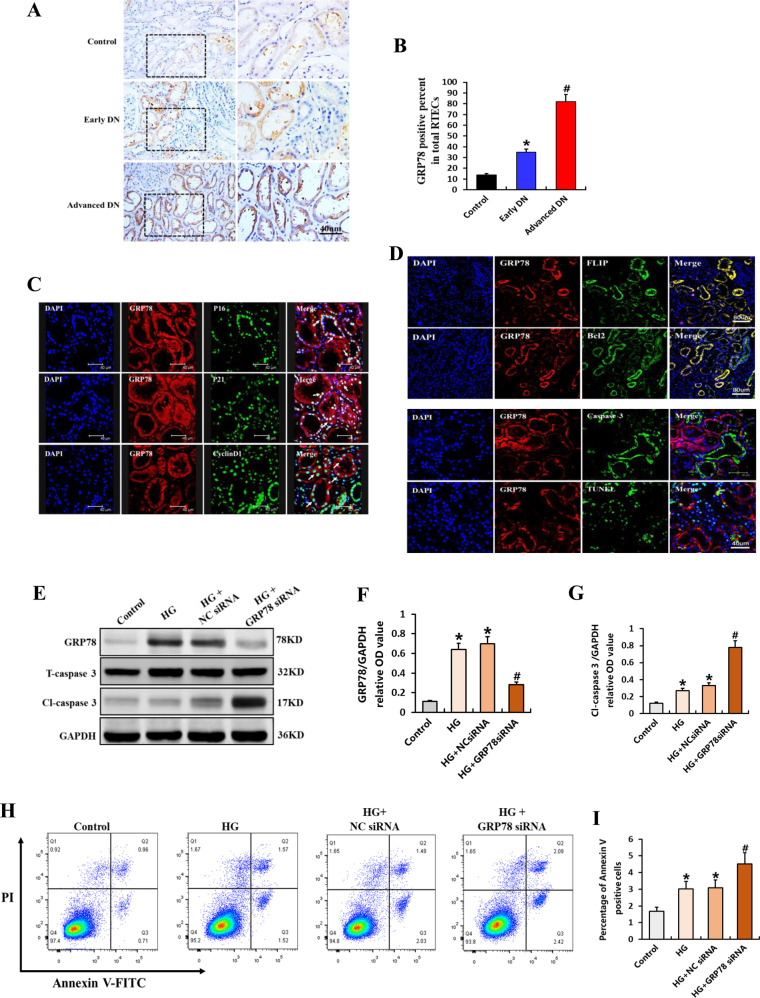
Role of GRP78 in generating the apoptosis-resistant phenotype of senescent RTECs in DN. **A** Representative immunostaining micrographs showing the expression of GRP78 in controls and patients with DN. Scale bar, 40 μm. **B** Quantification of the percentage of GRP78-positive RTECs. *P < 0.05 versus control; ^**#**^*P* < 0.05 versus early DN. **C** Representative immunofluorescence staining for GRP78 (red) and p16, p21, cyclin D1 (green) in patients with DN. Arrows indicate co-expression in the same tubules. Scale bar, 40 μm. **D** Immunofluorescence staining for GRP78 (red) and FLIP and Bcl2 (Scale bar, 80 μm); GRP78 and caspase 3, TUNEL (Scale bar, 40 μm). **E** Western blotting results for the expression of GRP78, total caspase 3, and cleaved caspase 3 in RTECs. **F**, **G** Expression levels quantified relative to GAPDH in different groups (*n* = 4) *P < 0.05 versus control; ^**#**^*P* < 0.05 versus HG. **H**, **I** Apoptosis rate as detected by flow cytometry and the percentage of Annexin V-positive RTECs (*n* = 6). Data are expressed as the mean ± SD for each group. *P < 0.05 versus control; ^#^*P* < 0.05 versus HG.

Previous studies revealed that GRP78 is involved in the progression of DN and acts as an important mediator of apoptotic resistance in several types of tumors [13, 34, 36]. To explore whether GRP78 also contributes to apoptotic resistance in senescent RTECs in the context of DN, we examined GRP78 expression and its relationships with antiapoptotic phenotypes in clinical DN samples, mice with STZ-induced DN, and HG-treated RTECs. As shown in Fig. 6A, B, GRP78 was mainly expressed in RTECs, and the percentage of GRP78 expression was higher in patients with DN than in controls, reaching unregulated levels in advanced DN tissues. Recent studies have revealed that GRP78 mediates RTECs senescence [13, 34]. Consistent with this finding, GRP78 was co-expressed with p16, p21, and cyclin D1 in patients with DN (Fig. 6C). Moreover, GRP78 exhibited co-expression with antiapoptotic proteins (FLIP and Bcl-2) but was rarely co-expressed with caspase 3 or TUNEL (Fig. 6D). Transfection of GRP78-siRNA significantly upregulated the expression of cleaved caspase 3 in vitro (Fig. 6E–G). Flow cytometry yielded similar results for RTECs with HG-induced apoptosis (Fig. 6H–I). These results suggest that GRP78 regulates apoptotic resistance in senescent RTECs in the context of DN.

**Fig. 7:**
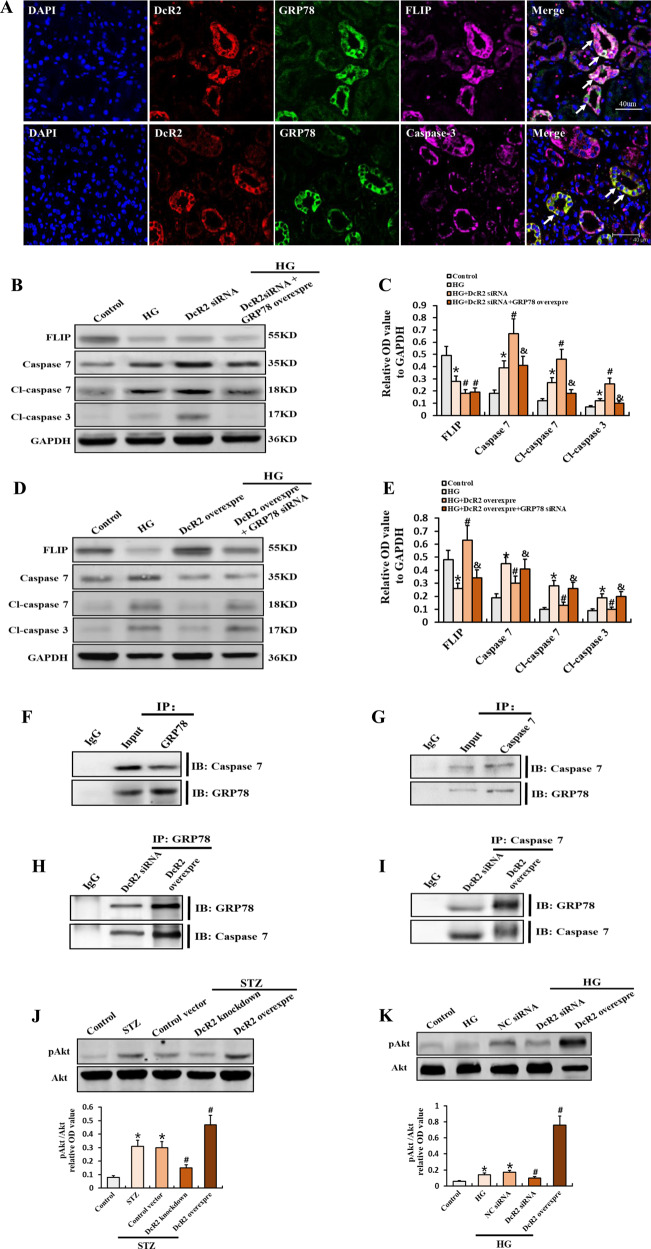
DcR2–GRP78 interactions mediated the apoptosis-resistant phenotype of senescent RTECs by regulating the antiapoptotic activity of GRP78. **A** Representative immunofluorescence staining for DcR2 (red), GRP78 (green), and FLIP or caspase 3 (purple) in patients with DN. Long arrows indicate co-localization in the same tubules. Scale bar, 40 μm. **B**, **D**. Western blotting results for FLIP, caspase 7, cleaved caspase 7, and cleaved caspase 3 expression in HG-induced RTECs. **C**, **E** Levels of each in B, D quantified in relation to GAPDH expression in different groups (*n* = 4). *P < 0.05 versus control; ^#^*P* < 0.05 versus HG; ^&^*P* < 0.05 versus HG + DcR2 siRNA/HG + DcR2 overexpression. **F**, **G** Reciprocal co-immunoprecipitation results for the interaction of GRP78 and caspase 7 in STZ-DN mice (*n* = 6). **H**, **I** The interaction of GRP78 and caspase 7 in HG-induced RTECs after transfection of DcR2 siRNA or overexpression plasmids. **J**, **K**. Western blotting results for the expression of Akt and pAkt and the relative levels of pAkt to Akt quantified in different groups (*n* = 4). *P < 0.05, versus control; ^#^*P* < 0.05, versus STZ/HG. Data are expressed as mean ± SD for each group.

To determine the role of DcR2–GRP78 interactions in promoting apoptotic resistance in senescent RTECs, we first examined levels of DcR2, GRP78, FLIP, and caspase 3 expression in patients with DN. Figure 7A shows that DcR2 and GRP78 mainly co-localized with the antiapoptotic protein FLIP rather than caspase 3. Subsequently, GRP78 knockdown significantly decreased DcR2 overexpression-exhibited antiapoptotic effects in HG-primed RTECs, as evidenced by increase in cleaved caspase 3 expression and decrease in FLIP expression. However, GRP78 overexpression produced the opposite effects (Fig. 7B–E). Together, these results indicate that the interaction between DcR2 and GRP78 mediates apoptotic resistance in senescent RTECs.

### DcR2 enhances the antiapoptotic activity of GRP78

Previous studies have indicated that GRP78 promotes the survival of cancer cells by exerting antiapoptotic effects via interactions with caspase 7 in the endoplasmic reticulum (ER) and activation of pro-proliferative PI3K/Akt signaling in the cell membrane [37–39]. Therefore, we first investigated whether GRP78 interacts with caspase 7 in STZ-DN mice. As shown in Fig. 7F, G, the reciprocal co-IP analysis demonstrated that GRP78 binds to caspase 7. Consistently, GRP78 overexpression remarkably decreased DcR2 siRNA-induced caspase 7 activity in HG-primed RTECs, as evidenced by the attenuated expression of cleaved caspase 7 (Fig. 7B, C). GRP78 knockdown reversed these effects (Fig. 7D, E). We then explored the effects of DcR2 on the interaction between GRP78 and caspase 7. The reciprocal co-IP analysis indicated that DcR2 overexpression enhanced the interaction between GRP78 and caspase 7 in HG-induced RTECs (Fig. 7H, I). The uncropped western blots and protein ladders were shown in supplementary Fig. S2. In contrast, DcR2 siRNA decreased binding of GRP78 to caspase 7 (Fig. 7H, I).

Additionally, we investigated whether DcR2 affects Akt activation. DcR2 overexpression increased Akt phosphorylation in mice STZ-induced DN, although these effects were attenuated by DcR2 knockdown (Fig. 7J). Similar results were observed in HG-treated RTECs (Fig. 7K). Collectively, these data suggest that DcR2 not only promotes the interaction between GRP78 and caspase 7 but also enhances Akt phosphorylation, indicating that DcR2 mediates apoptotic resistance in senescent RTECs by promoting the antiapoptotic activity of GRP78.

## Discussion

**Fig. 8:**
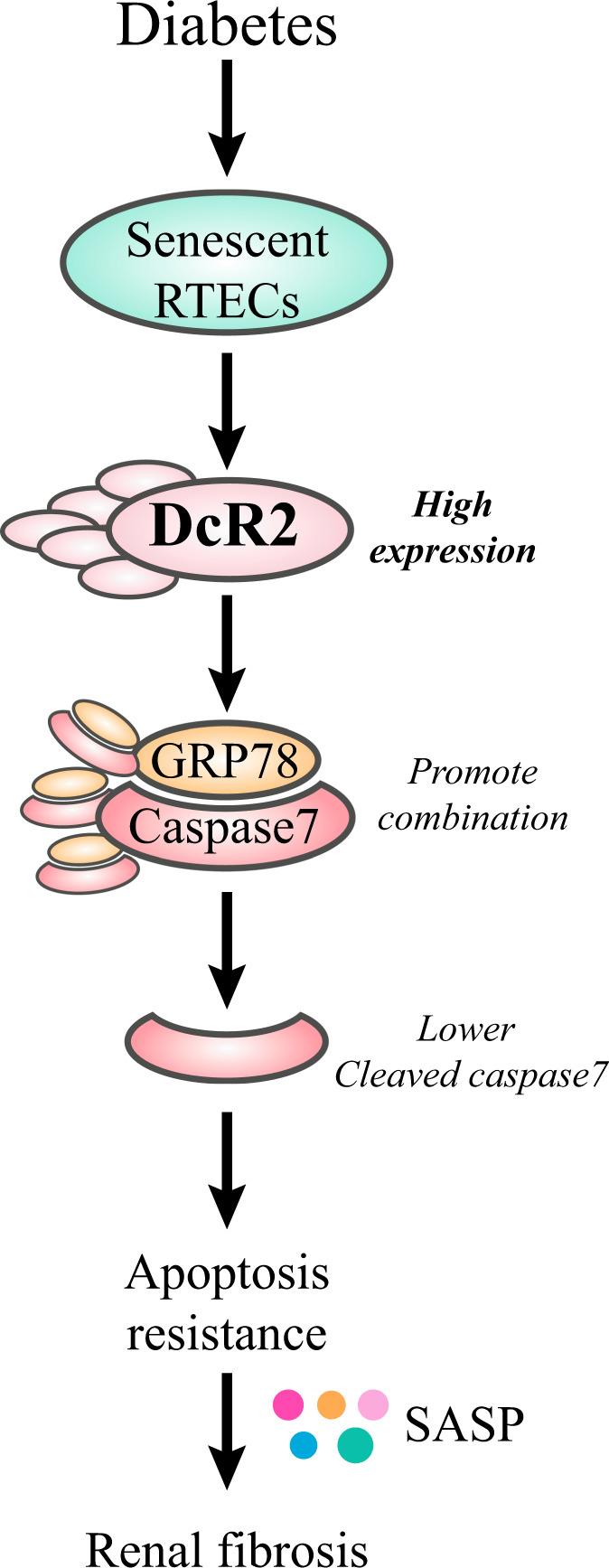
Schematic showing how DcR2–GRP78 may mediate the apoptosis-resistant phenotype of senescent tubular cells and renal fibrosis in DN. DcR2 is highly expressed in senescent renal tubular epithelial cells (RTECs) and mediates the apoptosis-resistant phenotype of senescent RTECs and renal fibrosis by interacting with GRP78, thereby contributing to progression of DN. DcR2 promotes the apoptosis-resistant activity of GRP78, leading to caspase 7 binding and Akt phosphorylation, which in turn result in persistent accumulation of senescent cells and SASP secretion, ultimately accelerating renal fibrosis.

In the present study, we investigated the contribution of DcR2 to apoptotic resistance in senescent RTECs. DcR2-positive senescent RTECs exhibited apoptosis-resistant phenotypes in biopsy samples from patients with DN and in mice with STZ-induced DN. Moreover, DcR2 mediated the apoptotic resistance of senescent RTECs and diabetic renal fibrosis in vitro and in vivo. Quantitative proteomics and validation studies suggested that this occurs via the direct interaction of DcR2 with GRP78. Specifically, our results suggest that DcR2 mediates apoptotic resistance by enhancing the GRP78–caspase 7 interaction and promoting Akt activation, as shown in Fig. 8.

Accumulating evidence suggests that RTEC senescence is an early cellular event in diabetes, playing an essential role in the progression of DN [8, 23]. Our earlier research revealed that DcR2 is scarcely expressed in healthy adult kidney tissue but is considerably upregulated in patients with DN and was positively associated with renal fibrosis scores and kidney dysfunction [14]. Furthermore, DcR2 exhibited co-expression with senescent markers including p16, p21, and SA-β-gal in patients with DN [20]. The results of our model experiments using STZ-DN mice are in accordance with these findings, supporting the notion that DcR2 promotes renal fibrosis by mediating the senescent phenotype of RTECs in vivo [15].

DcR2, a transmembrane receptor of TNF-related apoptosis-inducing ligand, has also been associated with resistance to chemotherapy and tumor progression [30, 31, 40]. In our recent study, DcR2 was identified as a specific marker of senescence in RTECs, with further research indicating that DcR2 mediates diabetic renal fibrosis by enhancing senescent phenotypes in RTECs [15]. In the present study, DcR2-positive senescent RTECs exhibited apoptotic resistance characterized by increased expression of antiapoptotic proteins (FLIP and Bcl2) and decreased expression of proapoptotic proteins (caspase 3, caspase 8, and TUNEL) in both patients with DN and mice with STZ-induced DN, consistent with our previous findings [20]. In vitro and in vivo experiments further verified that DcR2 mediates apoptotic resistance in senescent RTECs as well as diabetic renal fibrosis. Collectively, these findings suggest that DcR2 is not only a specific marker of senescence in RTECs but also mediates the apoptotic resistance of senescent RTECs, ultimately contributing to the pathogenesis of DN. Targeting DcR2, therefore, represents a promising therapeutic strategy for attenuating the accumulation of senescent cells and preventing the progression of DN.

Proteomic and pathway analyses suggested that pathways related to apoptosis, DNA replication, and other key processes are involved in the pathogenesis of DN. Since the renal tissues used for proteomic analyses were obtained from patients with DN, the samples contained many mesangial cells, inflammatory cells, and fibroblasts in addition to senescent RTECs. Therefore, the pathway analysis may have yielded some data that are not consistent with the characteristics of senescent cells. For example, the pathways associated with DNA replication identified in our analyses may be involved in the proliferation of mesangial cells, inflammatory cells, and fibroblasts, which may, in turn, contribute to glomerular injury, inflammation, and fibrosis, respectively. In addition, pathway analyses enriched for apoptosis-related proteins will include both pro-apoptotic and antiapoptotic proteins. In this study, enrichment of GRP78 was related to apoptotic resistance. Our quantitative proteomics and co-IP experiments involving samples from patients with DN, STZ-DN mice, and HG-treated RTECs support the notion that DcR2 interacts directly with GRP78. GRP78 is expressed primarily in the ER of mammalian cells, where it mediates the unfolded protein response by maintaining the stress sensors of the ER (PERK, ATF6, and IRE1) in an inactive form [41, 42]. Our previous studies demonstrated that advanced glycation end products promote the premature senescence of RTECs by activating GRP78-dependent ATF4/p16 and p21 signaling in DN [13, 34]. Consistent with these findings, we observed co-expression of GRP78 and several markers of senescence (p16, p21, and CyclinD1) in STZ-DN mice. Research has further indicated that GRP78 is implicated in enhancing tumor proliferation and metastasis and contributes to drug resistance by suppressing stress-induced cellular apoptosis [43–45]. Targeting GRP78 with small molecular or specific binding peptides inhibits growth and leads to the apoptosis of cancer cells [46]. The present findings demonstrate that DcR2 mediates apoptotic resistance in senescent RTECs by interacting with GRP78, resulting in persistent accumulation of senescent RTECs and SASP, thereby accelerating renal fibrosis in animal models and patients with DN.

GRP78, a potential target in cancer therapy, promotes stress-induced apoptotic resistance and tumor progression by interacting with pro-apoptotic factors, such as caspase 7 and Bik [38, 47]. Previous studies have revealed that the interaction of Kringle 5 with GPR78 at the cell surface contributes to caspase 7 activation and that Kringle 5 exposure can activate both caspase 3 and caspase 8 in endothelial cells [48–50]. Moreover, the interaction of tumor suppressor prostate apoptosis response-4 with GRP78 at the cell surface has been shown to induce apoptosis of prostate tumor cells via ER stress and activation of the caspase 8/3 pathway [47]. Our experiments involving tissues from mice with STZ-induced DN and HG-treated RTECs add to this knowledge, demonstrating that DcR2 mediates the apoptotic resistance of senescent RTECs by promoting the interaction of GRP78 with caspase 7.

Although GRP78 is mainly expressed in the lumen of the ER, it exhibits putative plasma membrane domains and is located in both cellular and ER membranes [51]. GRP78 expressed at the cell surface promotes the proliferation, migration, and apoptosis of cancer cells by modulating cellular signaling pathways, particularly the PI3K/Akt pathway [52]. In the current study, we observed that DcR2 regulates Akt phosphorylation in vivo and in vitro, which may be attributable to the DcR2-mediated activation of GRP78 activity. Cell-surface GRP78 activates Akt signaling via binding of its N-terminal region to alpha-2 macroglobulin [53]. Based on the available evidence, we speculate that DcR2 may inhibit the degradation of caspase-7 from GRP78 in the ER, silence the caspase 8/3 pathway, and enhance alpha-2 macroglobulin-Akt signaling by binding to GRP78 at the cell surface, thereby inducing apoptotic resistance in senescent RTECs in the context of DN (Fig. 8). Previous studies have indicated that the stability of GRP78 is tightly regulated by the ubiquitin ligase GP78 and the deubiquitylase OTUD3 [54, 55]. In contrast to the downregulation of GRP78 stability and the suppression of tumor metastasis by GP78, OTUD3 enhances GRP78 stability, leading to the growth of lung cancer cells and contributing to tumorigenesis [54, 55]. However, further studies are required to determine whether DcR2 promotes GRP78 stability by regulating the balance between the activities of ubiquitin ligase and deubiquitylase.

In summary, the current findings demonstrate that DcR2 mediates the apoptosis-resistance phenotype of senescent RTECs and renal fibrosis by interacting with GRP78, thereby contributing to the pathogenesis of DN. Mechanistically, our experiments indicated that DcR2 specifically interacts with GRP78, enhances GRP78–caspase 7 interactions, and disturbs the balance between anti- and proapoptotic protein expression, ultimately leading to apoptosis resistance, accumulation of senescent cells, sustained SASP secretion, and renal fibrosis. These results suggest that DcR2 is a specific marker of apoptotic resistance in senescent RTECs and that targeting the DcR2–GRP78 axis to eliminate senescent RTECs is a promising therapeutic strategy for the prevention and treatment of DN.

## Methods and materials

### Patients

A total of 241 patients with biopsy-proven DN admitted to Daping Hospital (Chongqing, China) between January 2012 and December 2019 were enrolled in the study. The inclusion and exclusion criteria were consistent with those of our previously published studies [14, 56]. Patients were divided into early DN (*n* = 83) and advanced DN (*n* = 158) groups based on the urinary albumin-to-creatinine ratio and renal function (calculated using the MDRD equation) as determined using at least two randomized measurements [57]. Early DN was defined as a urinary albumin-to-creatinine ratio between 30 and 300 mg/g*Cr with an estimated glomerular filtration rate (eGFR) >90 mL/min/1.73 m^2^, whereas advanced DN was defined as a urinary albumin-to-creatinine ratio >300 mg/g with an eGFR <90 mL/min/1.73 m^2^. Eighteen control samples of healthy kidney tissue were obtained from the unaffected portion of the organ in patients undergoing tumor nephrectomies. Data related to demographic characteristics, blood biochemistry, urine albumin excretion, and renal function parameters were collected. The study protocol was approved by the Ethical and Protocol Review Committee of the Army Medical University, and informed consent was obtained from all participants.

### Animal models

Eight-week-old male C57BL/6J mice were randomly selected and injected intraperitoneally with STZ (50 mg/kg body weight; Sigma-Aldrich) as previously described [15]. Mice with blood glucose levels >250 mg/dL were selected and euthanized at 24 weeks old. A portion of each kidney was fixed with 10% formalin for histological and immunohistochemical staining. Another portion of each kidney was stored at −80 °C for RNA and protein extraction. The experimental protocols were approved by the Ethics Committee of Army Medical University (approval number: 2014-90).

### Gene transfer of DcR2-siRNA or DcR2-overexpression plasmid in vivo

DcR2-siRNA or DcR2-overexpression plasmids were constructed and purchased from Obio Technology (Shanghai, China). The detailed characteristics of the plasmids, ultrasound microbubble-mediated gene transfer technique, and transfer efficiency were the same as described in our previously published research [15, 33]. Briefly, DcR2 expression plasmids were mixed with SonoVue (Bracco, Milan, Italy) at a ratio of 1:1 (vol/vol). The mixed solution (400 μL) was injected via the tail vein 2 weeks after STZ injection. The ultrasound transducer (Therasonic, United Kingdom) was immediately applied to the kidney with a continuous wave output of 1 MHz at a power of 2 W/cm^2^ for 5 min on each side. For the control vector group, the same amount of empty control plasmid was used. Gene transfection was performed at 10, 12, 14, 16, 18, 20, and 22 weeks. All mice were euthanized at 24 weeks (experimental design, Fig. 2A).

### Histological and immunohistochemical staining

Masson trichrome staining was performed in accordance with a standard protocol and analyzed under an Olympus microscope (Tokyo, Japan). All samples were incubated with primary anti-DcR2 (ab108421; Abcam, Cambridge, UK), anti-α-SMA (BM0002; Boster Biotechnology, Wuhan, China), anti-collagen IV (ab111742; Abcam), and anti-GRP78 (ab21685; Abcam). At least ten fields were randomly selected to evaluate the percentage of positive RTEC staining.

### Immunofluorescence staining

All sections were incubated with anti-DcR2 antibody followed by Alexa-555 conjugated goat anti-rabbit antibody (ab150078; Abcam), FLIP (ab8421; Abcam), Bcl2 (ab182858; Abcam), and caspase 3 (ab184787; Abcam) followed by Alexa-647 conjugated monkey anti-goat antibody (ab150135; Abcam); α-SMA, collagen IV, GRP78, fibronectin (ab6328; Abcam), p16 (ab54210; Abcam), p21 (2947 S, Celling Signaling), cyclin D1 (ab16663; Abcam), IL-6 (bs-4587MM, Bioss, China), and TGF-β1 (ab179615; Abcam) incubated with Alexa-488 conjugated goat anti-mouse antibody (ab150117; Abcam) at 37 °C for 1 h and co-stained with DAPI (C1006; Biyuntian Biotechnology, China). In addition, renal sections from STZ-DN mice were incubated with the TUNEL fluorescein kit (Biyuntian Biotechnology) according to the manufacturer’s protocol. Images were obtained via confocal microscopy (Leica, Germany) and analyzed using Image J software (version 1.37; NIH, Bethesda, USA).

### Quantitative real-time PCR

Total RNA was extracted using TRIzol reagent (Invitrogen, Carlsbad, CA, USA) in accordance with the manufacturer’s instructions, and 1 μg total RNA was synthesized into cDNA in a 20 μL reaction buffer using a reverse transcription PCR kit (Takara, Kyoto, Japan). The primer sequences are shown in Supplementary Table S2.

### Western blot analysis

Western blot analysis was performed on renal lysates or cell extracts using the following primary antibodies: anti-DcR2 (ab108421; Abcam), anti-FLIP (ab8421; Abcam), anti-cleaved caspase 3 (ab214430; Abcam), anti-caspase 8 (ab25901; Abcam), caspase 3 (ab184787; Abcam), anti-caspase 7 (ab255818; Abcam), anti-cleaved caspase 7 (ab256469; Abcam), Akt (sc5298, Santa Cruz), pAkt (sc135650, Santa Cruz), and anti-GAPDH (BM3876; Boster). The intensity of each band was analyzed using Quantity One software (Bio-Rad, Hercules, CA, USA).

### Cell cultures and treatment

Primary mouse RTECs were isolated and cultured as described previously [20]. The second-passage cells were exposed to a high concentration of glucose (30 mmol/L) for 48 h to construct a model of cellular senescence. The single-cell suspensions were obtained from DcR2-positive and DcR2-negative cells using magnetic affinity cell sorting. The detailed methods are described in our previously published work [20]. Moreover, a polyplus transfection system was used to transfect second-passage RTECs with the control vector and the DcR2-overexpression, DcR2-siRNA, and GRP78-siRNA plasmids (Obio Technology).

### Detection of caspase-3 activity and the expression of caspase 3-GreenNuc™ and Annexin V-mCherry

Extracts containing 50 μg of protein were incubated with 100 μM of the enzyme-specific colorimetric substrate Ac-DEVD-pNA at 37 °C for 2 h. The colorimetric release of p-nitroaniline was determined by measuring the absorbance at 405 nm. In addition, cells were incubated with caspase 3-GreenNuc™ substrate and Annexin V-mCherry at room temperature for 30 min (C1077S; Biyuntian) and co-stained with DAPI (C1006; Biyuntian). Images were obtained using confocal microscopy (Leica, Germany).

### Flow cytometry analysis

The treated cells were harvested and stained using an Annexin V-PI Apoptosis Detection Kit (BioLegend, CA, USA). After 30 min of incubation in the dark, the cell apoptosis ratio was measured using a flow cytometer (BD Biosciences, San Jose, CA, USA).

### Renal tissue for IP-LC-MS/MS analysis

Eighteen patients with biopsy-proven DN and 18 healthy controls were enrolled. The clinical characteristics, inclusion and exclusion criteria, and detailed methods of IP combined with LC-MS/MS analysis were described in our previously published study [15]. Briefly, the lysed renal tissue was incubated overnight with 2 μg of anti-DcR2 antibody (2 μg) at 4 °C. A total of 40 μL protein A + G agarose (Bio-Rad Biotechnology) was added to the solution, which was incubated at 4 °C for 3 h. The collected sediments were then conjugated with a trypsin gel. The tryptic peptides were fractionated via LC, and each fraction was analyzed via LC/MS (Thermo Fisher Scientific, Waltham, MA, USA). The raw data were analyzed using MASCOT version 2.2 (Thermo Fisher Scientific). Three biological and technical replicates of each sample were performed to increase the confidence of the identified proteins. Proteins with an intensity ratio >2 (*p* < 0.05) from both biological replicates were considered differentially expressed DcR2-interacting proteins.

### Bioinformatics analysis

Pathway analysis of the differentially expressed DcR2-interacting proteins was performed using DAVID (Ver.6.8, https://david.ncifcrf.gov/) and PANTHER (Ver.9.0, http://www.pan therdb.org/) bioinformatics tools.

### Co-immunoprecipitation

Protein A magnetic beads (100 μL; Bio-Rad Biotechnology) were incubated with 5 μg DcR2 (ab108421; Abcam), Flag (ab205606; Abcam), and rabbit monoclonal IgG antibody (ab172730; Abcam) at room temperature for 1 h. Protein G magnetic beads (100 μL) were incubated with 5 μg GRP78 (ab212054; Abcam), Myc (ab32; Abcam), and mouse IgG antibody (ab131368; Abcam) at room temperature for 1 h. The above beads were washed and collected. In addition, the renal tissues and cells were lysed with ice-cold IP Lysis buffer on ice for 30 min and 10 min, respectively. The lysate was then mixed with the Protein A or G magnetic beads and incubated at 4 °C overnight to form the immune complex, following which 100 µL of SDS-PAGE loading buffer was added prior to incubation at 100 °C for 10 min. Finally, the samples were subjected to western blotting with anti-GRP78, anti-DcR2, anti-Myc, or anti-Flag antibody.

### Statistical analysis

Data are presented as the mean ± standard deviation (SD). Statistical differences were analyzed via one-way analysis of variance followed by multiple pairwise comparisons using the Newman–Keuls test or Dunnett’s T3 test. All analyses were performed using SPSS 18.0 software (SPSS Inc., Chicago, IL, USA). Statistical significance was set at *p* < 0.05.

## Supplementary information

aj-checklist

Supplementary tables

supplementary legends

supplementary Figure S1

Supplementary Figure S2

Certificate of editing

## Data Availability

All data generated or analyzed during this study are available from the first or corresponding author upon reasonable request.

## References

[1] Johansen KL, Chertow GM, Foley RN, Gilbertson DT, Herzog CA, Ishani A (2021). US renal data system 2020 annual data report: epidemiology of kidney disease in the United States. Am J Kidney Dis..

[2] Xu Y, Wang L, He J, Bi Y, Li M, Wang T (2013). Prevalence and control of diabetes in Chinese adults. JAMA..

[3] Qi R, Yang C (2018). Renal tubular epithelial cells: the neglected mediator of tubulointerstitial fibrosis after injury. Cell Death Dis.

[4] Hung PH, Hsu YC, Chen TH, Lin CL (2021). Recent Advances in diabetic kidney diseases: from kidney injury to kidney fibrosis. Int J Mol Sci.

[5] An Y, Xu F, Le W, Ge Y, Zhou M, Chen H (2015). Renal histologic changes and the outcome in patients with diabetic nephropathy. Nephrol Dial Transpl.

[6] Tchkonia T, Kirkland JL (2018). Aging, cell senescence, and chronic disease: emerging therapeutic strategies. JAMA..

[7] Di Micco R, Krizhanovsky V, Baker D, d’Adda, di Fagagna F (2021). Cellular senescence in ageing: from mechanisms to therapeutic opportunities. Nat Rev Mol Cell Biol.

[8] Santin Y, Lluel P, Rischmann P, Game X, Mialet-Perez J, Parini A (2020). Cellular senescence in renal and urinary tract disorders. Cells.

[9] Sturmlechner I, Durik M, Sieben CJ, Baker DJ, van Deursen JM (2017). Cellular senescence in renal ageing and disease. Nat Rev Nephrol.

[10] Munoz-Espin D, Canamero M, Maraver A, Gomez-Lopez G, Contreras J, Murillo-Cuesta S (2013). Programmed cell senescence during mammalian embryonic development. Cell..

[11] He S, Sharpless NE (2017). Senescence in Health and Disease. Cell..

[12] Tchkonia T, Zhu Y, van Deursen J, Campisi J, Kirkland JL (2013). Cellular senescence and the senescent secretory phenotype: therapeutic opportunities. J Clin Investig.

[13] Liu J, Huang K, Cai GY, Chen XM, Yang JR, Lin LR (2014). Receptor for advanced glycation end-products promotes premature senescence of proximal tubular epithelial cells via activation of endoplasmic reticulum stress-dependent p21 signaling. Cell Signal.

[14] Chen J, Zhang WW, Chen KH, Lin LR, Dai HZ, Li KL (2017). Urinary DcR2 is a novel biomarker for tubulointerstitial injury in patients with diabetic nephropathy. Am J Physiol Ren Physiol.

[15] Jia C, Ke-Hong C, Fei X, Huan-Zi D, Jie Y, Li-Ming W (2020). Decoy receptor 2 mediation of the senescent phenotype of tubular cells by interacting with peroxiredoxin 1 presents a novel mechanism of renal fibrosis in diabetic nephropathy. Kidney Int.

[16] Braun H, Schmidt BM, Raiss M, Baisantry A, Mircea-Constantin D, Wang S (2012). Cellular senescence limits regenerative capacity and allograft survival. J Am Soc Nephrol.

[17] Luo C, Zhou S, Zhou Z, Liu Y, Yang L, Liu J (2018). Wnt9a promotes renal fibrosis by accelerating cellular senescence in tubular epithelial cells. J Am Soc Nephrol.

[18] Jin J, Tao J, Gu X, Yu Z, Wang R, Zuo G (2017). P16 (INK4a) deletion ameliorated renal tubulointerstitial injury in a stress-induced premature senescence model of Bmi-1 deficiency. Sci Rep.

[19] Baker DJ, Childs BG, Durik M, Wijers ME, Sieben CJ, Zhong J (2016). Naturally occurring p16(Ink4a)-positive cells shorten healthy lifespan. Nature..

[20] Chen J, Chen KH, Fu BQ, Zhang W, Dai H, Lin LR (2017). Isolation and identification of senescent renal tubular epithelial cells using immunomagnetic beads based on DcR2. Exp Gerontol.

[21] Pignolo RJ, Passos JF, Khosla S, Tchkonia T, Kirkland JL (2020). Reducing senescent cell burden in aging and disease. Trends Mol Med.

[22] Deryabin PI, Shatrova AN, Borodkina AV (2021). Apoptosis resistance of senescent cells is an intrinsic barrier for senolysis induced by cardiac glycosides. Cell Mol Life Sci.

[23] Xu J, Zhou L, Liu Y (2020). Cellular senescence in kidney fibrosis: pathologic significance and therapeutic strategies. Front Pharm.

[24] Knoppert SN, Valentijn FA, Nguyen TQ, Goldschmeding R, Falke LL (2019). Cellular senescence and the kidney: potential therapeutic targets and tools. Front Pharm.

[25] Yosef R, Pilpel N, Tokarsky-Amiel R, Biran A, Ovadya Y, Cohen S (2016). Directed elimination of senescent cells by inhibition of BCL-W and BCL-XL. Nat Commun.

[26] Sanders YY, Liu H, Zhang X, Hecker L, Bernard K, Desai L (2013). Histone modifications in senescence-associated resistance to apoptosis by oxidative stress. Redox Biol.

[27] Kimberley FC, Screaton GR (2004). Following a TRAIL: update on a ligand and its five receptors. Cell Res.

[28] Collado M, Gil J, Efeyan A, Guerra C, Schuhmacher AJ, Barradas M (2005). Tumour biology: senescence in premalignant tumours. Nature..

[29] Sagiv A, Biran A, Yon M, Simon J, Lowe SW, Krizhanovsky V (2013). Granule exocytosis mediates immune surveillance of senescent cells. Oncogene..

[30] Vindrieux D, Reveiller M, Chantepie J, Yakoub S, Deschildre C, Ruffion A (2011). Down-regulation of DcR2 sensitizes androgen-dependent prostate cancer LNCaP cells to TRAIL-induced apoptosis. Cancer Cell Int.

[31] Lalaoui N, Morle A, Merino D, Jacquemin G, Iessi E, Morizot A (2011). TRAIL-R4 promotes tumor growth and resistance to apoptosis in cervical carcinoma HeLa cells through AKT. PLoS One.

[32] Ovadya Y, Krizhanovsky V (2018). Strategies targeting cellular senescence. J Clin Investig.

[33] Ju-Rong Y, Ke-Hong C, Kun H, Bi-Qiong F, Li-Rong L, Jian-Guo Z (2017). Transcription factor Trps1 promotes tubular cell proliferation after ischemia-reperfusion injury through cAMP-specific 3’,5’-cyclic phosphodiesterase 4D and AKT. J Am Soc Nephrol.

[34] Liu J, Yang JR, Chen XM, Cai GY, Lin LR, He YN (2015). Impact of ER stress-regulated ATF4/p16 signaling on the premature senescence of renal tubular epithelial cells in diabetic nephropathy. Am J Physiol Cell Physiol.

[35] Bian T, Tagmount A, Vulpe C, Vijendra KC, Xing C (2020). CXL146, a novel 4H-chromene derivative, targets GRP78 to selectively eliminate multidrug-resistant cancer cells. Mol Pharm.

[36] Lu G, Luo H, Zhu X (2020). Targeting the GRP78 pathway for cancer therapy. Front Med.

[37] Reddy RK, Mao C, Baumeister P, Austin RC, Kaufman RJ, Lee AS (2003). Endoplasmic reticulum chaperone protein GRP78 protects cells from apoptosis induced by topoisomerase inhibitors: role of ATP binding site in suppression of caspase-7 activation. J Biol Chem.

[38] Fu Y, Li J, Lee AS (2007). GRP78/BiP inhibits endoplasmic reticulum BIK and protects human breast cancer cells against estrogen starvation-induced apoptosis. Cancer Res.

[39] Fu Y, Wey S, Wang M, Ye R, Liao CP, Roy-Burman P (2008). Pten null prostate tumorigenesis and AKT activation are blocked by targeted knockout of ER chaperone GRP78/BiP in prostate epithelium. Proc Natl Acad Sci USA.

[40] Kretz AL, Trauzold A, Hillenbrand A, Knippschild U, Henne-Bruns D, von Karstedt S (2019). TRAILblazing strategies for cancer treatment. Cancers.

[41] Lee AS (2014). Glucose-regulated proteins in cancer: molecular mechanisms and therapeutic potential. Nat Rev Cancer.

[42] Ni M, Zhou H, Wey S, Baumeister P, Lee AS (2009). Regulation of PERK signaling and leukemic cell survival by a novel cytosolic isoform of the UPR regulator GRP78/BiP. PLoS One.

[43] Qian Y, Wong CC, Xu J, Chen H, Zhang Y, Kang W (2017). Sodium channel subunit SCNN1B suppresses gastric cancer growth and metastasis via GRP78 degradation. Cancer Res.

[44] Lee HK, Xiang C, Cazacu S, Finniss S, Kazimirsky G, Lemke N (2008). GRP78 is overexpressed in glioblastomas and regulates glioma cell growth and apoptosis. Neuro Oncol.

[45] Zheng HC, Takahashi H, Li XH, Hara T, Masuda S, Guan YF (2008). Overexpression of GRP78 and GRP94 are markers for aggressive behavior and poor prognosis in gastric carcinomas. Hum Pathol.

[46] Kang J, Zhao G, Lin T, Tang S, Xu G, Hu S (2013). A peptide derived from phage display library exhibits anti-tumor activity by targeting GRP78 in gastric cancer multidrug resistance cells. Cancer Lett.

[47] Burikhanov R, Zhao Y, Goswami A, Qiu S, Schwarze SR, Rangnekar VM (2009). The tumor suppressor Par-4 activates an extrinsic pathway for apoptosis. Cell..

[48] Davidson DJ, Haskell C, Majest S, Kherzai A, Egan DA, Walter KA (2005). Kringle 5 of human plasminogen induces apoptosis of endothelial and tumor cells through surface-expressed glucose-regulated protein 78. Cancer Res.

[49] Gu X, Yao Y, Cheng R, Zhang Y, Dai Z, Wan G (2011). Plasminogen K5 activates mitochondrial apoptosis pathway in endothelial cells by regulating Bak and Bcl-x(L) subcellular distribution. Apoptosis..

[50] Nguyen TM, Subramanian IV, Kelekar A, Ramakrishnan S (2007). Kringle 5 of human plasminogen, an angiogenesis inhibitor, induces both autophagy and apoptotic death in endothelial cells. Blood..

[51] Zhang Y, Liu R, Ni M, Gill P, Lee AS (2010). Cell surface relocalization of the endoplasmic reticulum chaperone and unfolded protein response regulator GRP78/BiP. J Biol Chem.

[52] Arap MA, Lahdenranta J, Mintz PJ, Hajitou A, Sarkis AS, Arap W (2004). Cell surface expression of the stress response chaperone GRP78 enables tumor targeting by circulating ligands. Cancer Cell.

[53] Gonzalez-Gronow M, Cuchacovich M, Llanos C, Urzua C, Gawdi G, Pizzo SV (2006). Prostate cancer cell proliferation in vitro is modulated by antibodies against glucose-regulated protein 78 isolated from patient serum. Cancer Res.

[54] Chang YW, Tseng CF, Wang MY, Chang WC, Lee CC, Chen LT (2016). Deacetylation of HSPA5 by HDAC6 leads to GP78-mediated HSPA5 ubiquitination at K447 and suppresses metastasis of breast cancer. Oncogene..

[55] Du T, Li H, Fan Y, Yuan L, Guo X, Zhu Q (2019). The deubiquitylase OTUD3 stabilizes GRP78 and promotes lung tumorigenesis. Nat Commun.

[56] Chen K, Dai H, Yuan J, Chen J, Lin L, Zhang W (2018). Optineurin-mediated mitophagy protects renal tubular epithelial cells against accelerated senescence in diabetic nephropathy. Cell Death Dis.

[57] Fan Y, Yi Z, D’Agati VD, Sun Z, Zhong F, Zhang W (2019). Comparison of kidney transcriptomic profiles of early and advanced diabetic nephropathy reveals potential new mechanisms for disease progression. Diabetes..

