# Supplementary material for: Decoy receptor 2 mediates the apoptosis-resistant phenotype of senescent renal tubular cells and accelerates renal fibrosis in diabetic nephropathy
Source: Cell Death Dis. 2022 Jun 3;13(6):522. doi: 10.1038/s41419-022-04972-w (PMC9166763; doi:10.1038/s41419-022-04972-w)
Supplement: Supplementary file 2 — Supplementary tables [file 41419_2022_4972_MOESM2_ESM.docx]

**Supplementary Table S1. Demographic and clinical characteristics of patients with DN and normal control**

|  | Control | Early DN | Advanced DN |
| --- | --- | --- | --- |
| Numbers | 18 | 83 | 158 |
| Age (years) | 57.5±8.6 | 60.5±9.6 | 58.4±8.9 |
| Sex, M/F | 9/9 | 43/40 | 86/72 |
| Duration of DN (years) | — | 7.2±4.8 | 11.5±5.7 |
| BMI (kg/m^2^) | 23.8±1.0 | 24.8±2.1 | 25.4±3.3 |
| SBP (mmHg) | 126**±**14 | 132**±**16 | 149**±**21**^*^** |
| DBP (mmHg) | 82±10 | 84±12 | 86±15 |
| HbA1c (%) | 5.7±0.6 | 7.6±0.5**^*^** | 8.3±1.4**^*^** |
| ACR (mg/gCr) | 5.8±3.6 | 124.3±33.7**^*^** | 2384.3±584.6**^*&^** |
| eGFR (mL/min/1.73 m^2^) | 117.4±26.4 | 113.1±9.4 | 64.1±14.5**^*&^** |

BMI, body mass index; SBP, systolic blood pressure; DBP, diastolic blood pressure; HbA1c, hemoglobin A1c; ACR, albumin to creatinine ratio; eGFR, estimated glomerular filtration rate.

*P < 0.05 versus control, ^&^P < 0.05 versus early DN.

**Supplementary Table S2. The primer sequences used for RT-PCR analysis**

| **Gene** | **Primer Sequence 5’ to 3’** | |
| --- | --- | --- |
|  | **Forward** | **Reverse** |
| DcR2 | AAATGTCCCGCTGGTGAATAC | GGCGGCACGATTCTGGAAA |
| a-SMA | TGCTGACAGAGGCACCACTGAA | CAGTTGTACGTCCAGAGGCATAG |
| Collagen I | CCTCAGGGTATTGCTGGACAAC | CAGAAGGACCTTGTTTGCCAGG |
| FLIP | CCGCAGGCTAACTTTCC | CCCATCCCACTCAACAAC |
| Caspase 8 | AAAGCGAAGCAGCCTATG | GCACCGAAGTCAGAATGAA |
| GAPDH | AGGTCGGTGTGAACGGATTTG | GGGGTCGTTGATGGCAACA |
