# Supplementary material for: Decoy receptor 2 mediates the apoptosis-resistant phenotype of senescent renal tubular cells and accelerates renal fibrosis in diabetic nephropathy
Source: Cell Death Dis. 2022 Jun 3;13(6):522. doi: 10.1038/s41419-022-04972-w (PMC9166763; doi:10.1038/s41419-022-04972-w)
Supplement: Supplementary file 3 — supplementary legends [file 41419_2022_4972_MOESM3_ESM.docx]

**SUPPLEMENTARY INFORMATION**

**Supplementary Table S1. Demographic and clinical characteristics of patients with DN and controls with healthy kidneys.**

**Supplementary Table S2. The primer sequences used for RT-PCR analysis.**

**Supplementary Figure S1. DcR2 expression in the renal tissue of STZ-treated mice after ultrasound microbubble-mediated gene transfer of DcR2-siRNA or DcR2-overexpression plasmid. A**. GFP expression in renal tissue on day 7 after gene transfection with control vector and DcR2-related plasmids (n=6 for each group). Scale bar, 160 μm. **B**. The level of renal DcR2 mRNA was detected via qPCR (n=6 for each group). **C–D**. DcR2 protein expression was measured via western blotting and quantified relative to levels of GAPDH expression (n=6 for each group). Data are expressed as the mean ± SD for each group. *P < 0.05 versus control; ^#^P < 0.05 versus STZ.

**Supplementary Figure S2. The uncropped western blots and protein ladders used for the analyses of GRP78 and procaspase 7 interaction.**
