# Supplementary figures and images for: Decoy receptor 2 mediates the apoptosis-resistant phenotype of senescent renal tubular cells and accelerates renal fibrosis in diabetic nephropathy
Source: Cell Death Dis. 2022 Jun 3;13(6):522. doi: 10.1038/s41419-022-04972-w (PMC9166763; doi:10.1038/s41419-022-04972-w)

**A**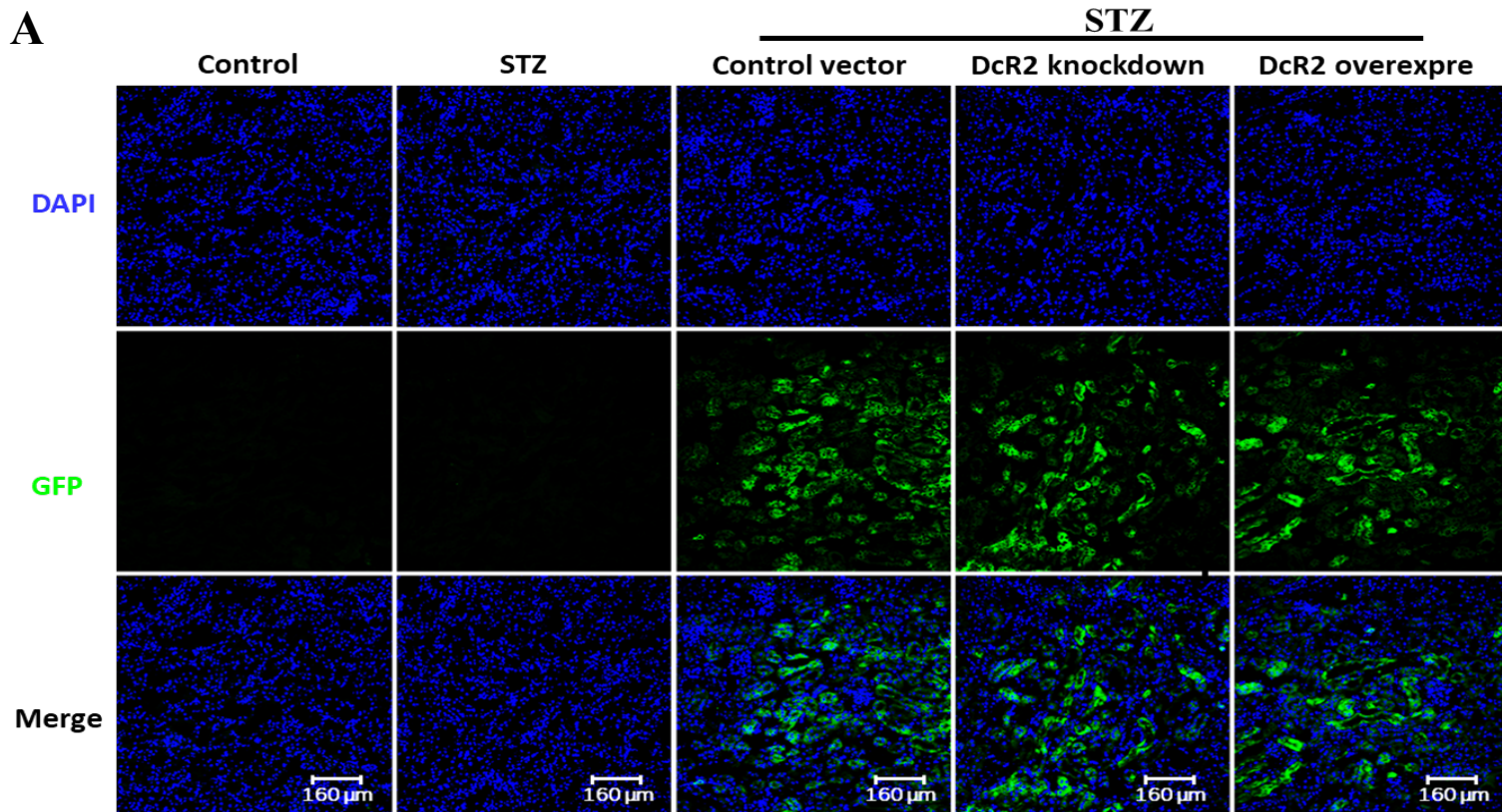**B**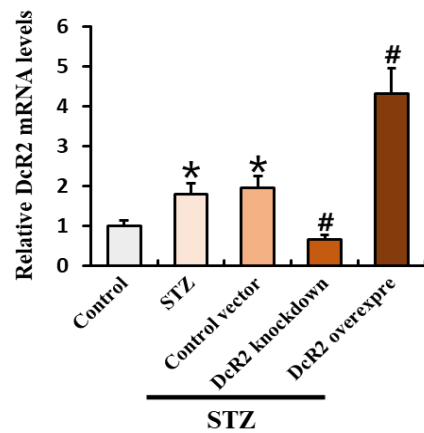**C**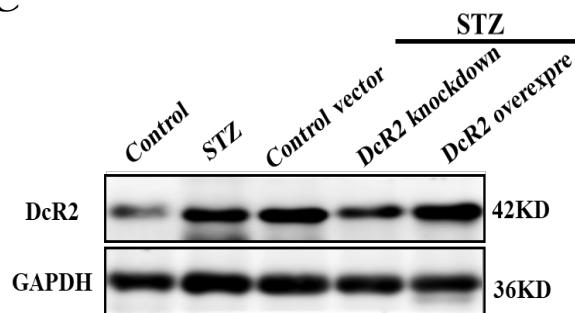**D**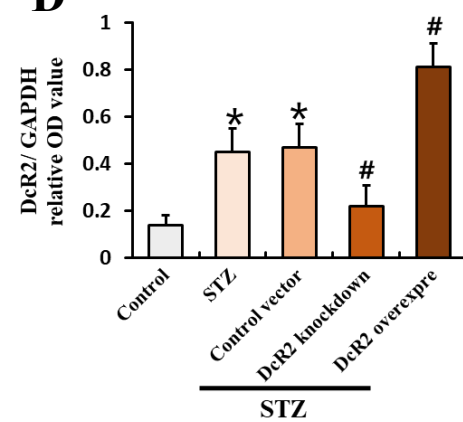

Supplement: Supplementary file 4 — supplementary Figure S1 [file 41419_2022_4972_MOESM4_ESM.pdf]

**Figure 7H**

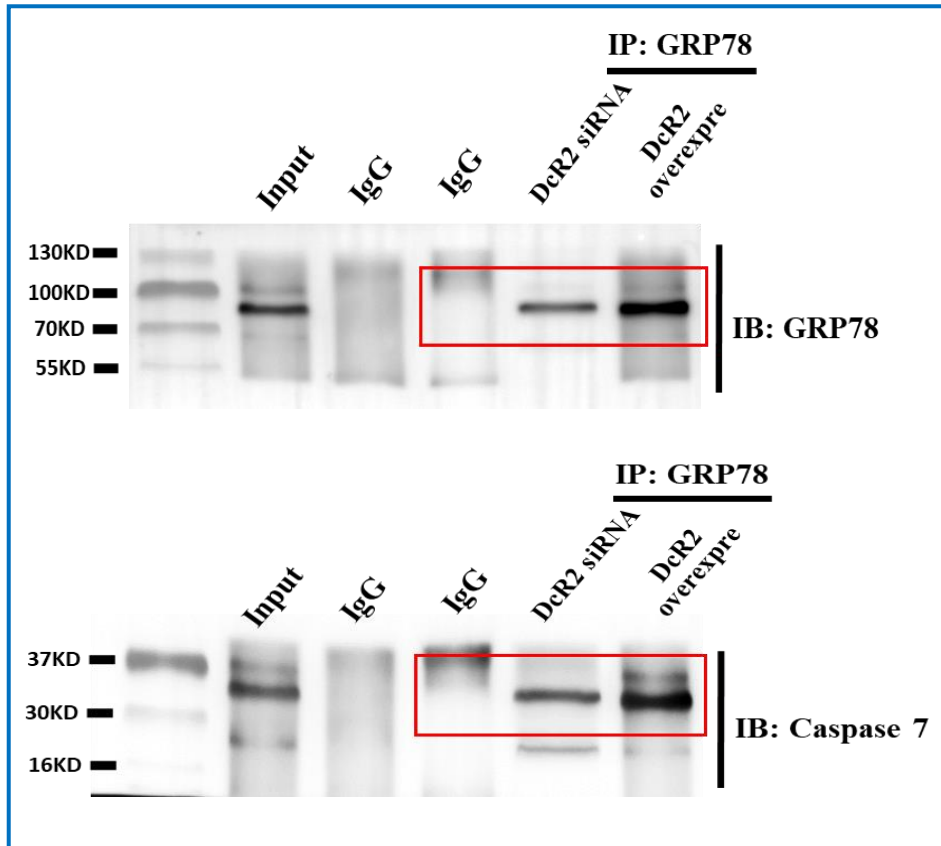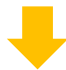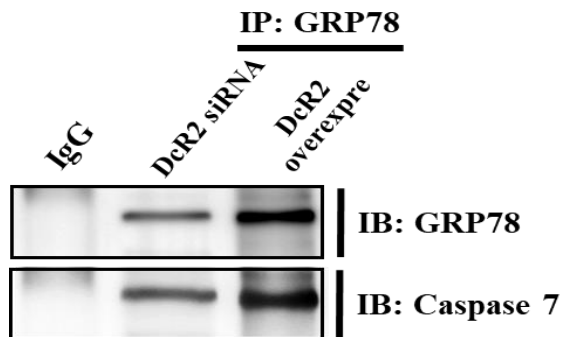

**Figure 7I**

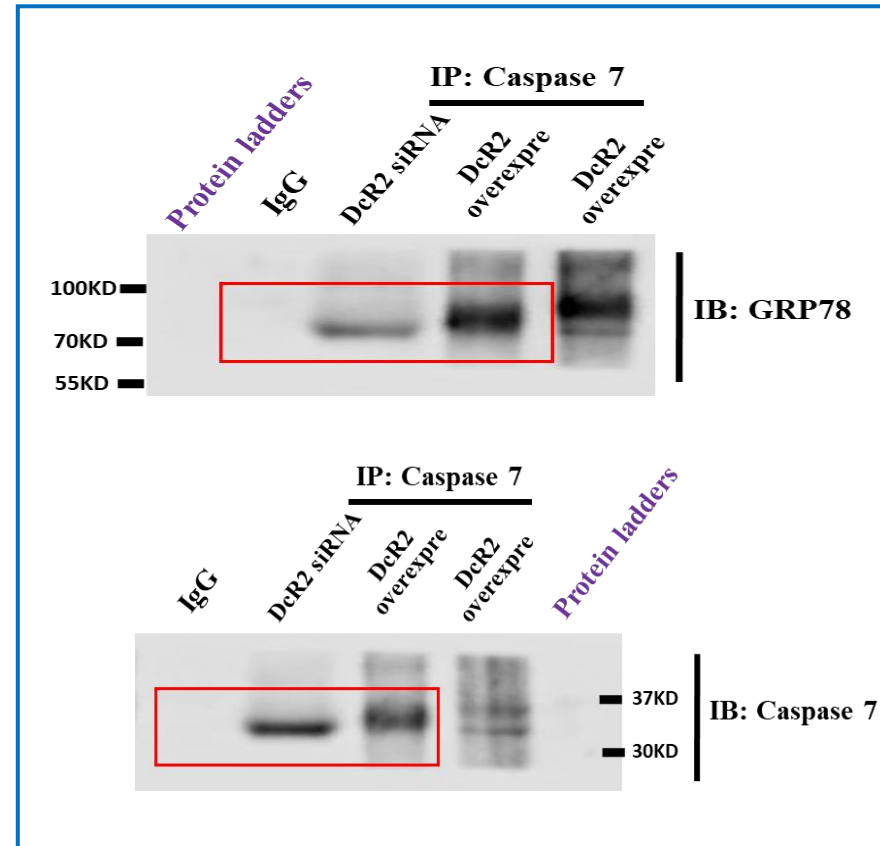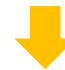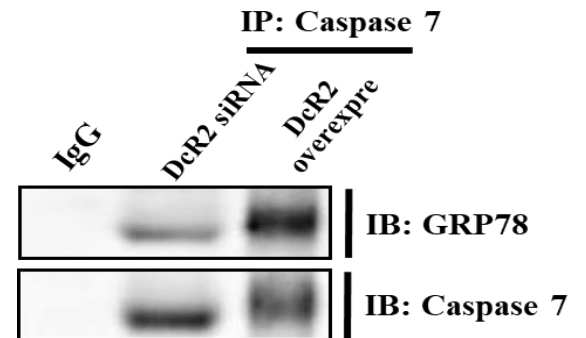

Supplement: Supplementary file 5 — Supplementary Figure S2 [file 41419_2022_4972_MOESM5_ESM.pdf]
